# Regional Alliance for Cervical Cancer Prevention in Eastern Europe and Central Asia: Progressing Towards the Target 1 of the Global Strategy for Cervical Cancer Elimination

**DOI:** 10.3390/healthcare13101209

**Published:** 2025-05-21

**Authors:** Silvia Ussai, Teymur Seyidov, Tamar Khomasuridze, on behalf of the Regional Alliance Group of Experts on HPV Vaccination

**Affiliations:** United Nation Population Fund, Eastern Europe and Central Asia, Regional Office, Istanbul 34349, Turkey; seyidov@unfpa.org (T.S.); khomasuridze@unfpa.org (T.K.)

**Keywords:** cervical cancer, eastern Europe and central Asia, regional alliance for cervical cancer prevention, HPV vaccination

## Abstract

**Background and Objectives:** Cervical cancer remains a critical public health challenge in Eastern Europe and Central Asia (EECA), where systemic barriers have hindered prevention efforts. This descriptive regional study evaluates progress toward achieving Target 1 of the WHO Global Strategy for Cervical Cancer Elimination—vaccinating 90% of girls by age 15—in 17 countries and territories. The research is situated within the context of the Regional Alliance for Cervical Cancer Prevention in EECA, a multi-stakeholder platform launched by UNFPA in 2021 to accelerate regional progress toward WHO targets. The Alliance supports countries through technical collaboration, shared learning, and political engagement. Therefore, as a secondary endpoint, the study explores possible correlations between national achievements and the post-2021 scale-up efforts supported by the Alliance. **Methods:** A standardized questionnaire, consolidated by United Nation Population Fund (UNFPA) technical experts, was disseminated in November 2024 to 17 national focal points, yielding 19 responses due to disaggregated submissions from Bosnia and Herzegovina. The survey collected data on HPV vaccination policies, delivery models, vaccine type, target populations, and coverage. **Results:** By late 2024, six countries had implemented HPV vaccination before 2021, while six more launched programs after the Regional Alliance’s formation in 2021. Coverage varied widely, from 0.2% in Brčko District to 99.3% in Uzbekistan. Most countries targeted girls aged 9–14, with increasing male inclusion and catch-up cohorts. Gardasil 4 was the most used vaccine, though Gardasil 9 is gaining ground. School-based and hybrid delivery strategies were associated with significantly higher coverage (*p* = 0.0121). Inferential analysis also showed significant variation by vaccine type (*p* = 0.0067) and a positive correlation with program maturity (ρ = 0.52, *p* = 0.067). However, findings should be interpreted considering limitations including reliance on self-reported country data and absence of independent validation. **Conclusions:** The results offer actionable insights into delivery models, gender inclusion, and regional disparities—supporting efforts to close the gap toward 2030 elimination targets in EECA Countries.

## 1. Introduction

Cervical cancer remains a significant public health challenge across Eastern Europe and Central Asia (EECA), with recent data showing that the region continues to face disproportionately high incidence and mortality rates compared to Western Europe. Despite global advances in cervical cancer prevention, the EECA region lags behind, primarily due to structural barriers in healthcare systems, limited access to services, and gaps in national public health strategies [1].

The primary oncogenic strains responsible for cervical cancer, HPV types 16 and 18, are widely prevalent in the region [2]. However, national responses have varied, with uneven implementation of HPV vaccination and organized screening programs. A 2023 baseline analysis from the WHO Global Cervical Cancer Elimination Initiative highlights both the heterogeneity in vaccine rollout and the low overall coverage in many EECA countries [3].

Recent population-based survey data confirm the fragmented nature of cervical cancer screening systems in the EECA region, with wide variation in program structure, implementation, and population coverage across nine countries, and many relying primarily on opportunistic rather than organized screening approaches. Screening coverage among women aged 30 to 49 remains below 50% in several countries, and disparities are further compounded by cultural barriers, lack of public awareness, and healthcare system distrust [4].

Efforts to establish standardized screening guidelines and invest in healthcare infrastructure have been constrained by financial and logistical limitations. Workforce shortages, weak data systems, and regional inequalities remain major hurdles. Although economic evaluations consistently show the cost-effectiveness of integrated vaccination and screening programs, implementation remains incomplete.

To address these challenges, the World Health Organization (WHO)’s global strategy for cervical cancer elimination has set specific pillars to reduce the disease as a public health problem through a comprehensive, prevention-focused approach [5]. Launched in 2020, the strategy outlines three core targets to be achieved by 2030: vaccinating 90% of girls by age 15 against human papillomavirus (HPV), screening 70% of women with a high-performance test at least twice in their lifetime, and ensuring that 90% of women identified with cervical disease receive appropriate treatment. This triple-intervention model (90-70-90) is designed to prevent new infections, detect precancerous changes early, and treat cases effectively—thereby accelerating progress toward the long-term goal of reducing cervical cancer incidence below four cases per 100,000 women globally.

The Regional Alliance for Cervical Cancer Prevention in EECA (*the “Alliance”*), launched by United Nation Population Fund (UNFPA) in 2021, emerged as a multi-stakeholder platform designed to promote cross-country collaboration and technical alignment with WHO goals. The Alliance brings together national health authorities, international organizations, civil society, and academic institutions to accelerate progress in the region [6].

By exploring HPV program progress and the contribution of the Alliance to the regional coordination towards Target 1 of the WHO strategy, this study aims to shed light on both achievements and ongoing gaps in the pursuit of cervical cancer elimination in EECA.

## 2. Materials and Methods

This descriptive study utilized a standardized survey questionnaire developed by the UNFPA Regional Office to collect country-level data on HPV vaccination efforts in Eastern Europe and Central Asia. The instrument is reproduced in Table 1, presenting a summary of national-level responses based on this tool.

The results of Table 1 were organized into two panels to improve clarity and facilitate comparative analysis. Table 2 presents an overview of national HPV vaccination program characteristics, grouped by EECA subregions (Eastern Europe, South Caucasus, Western Balkans, and Central Asia), including program start year, vaccine type, and delivery model and provides detailed country-specific information on target age groups and reported vaccination coverage, sorted alphabetically for readability.

The questionnaire was designed to ensure consistency, comparability, and completeness across participating countries and territories. It was informed by existing WHO/UNFPA technical tools, including the HPV Vaccination Introduction Readiness Assessment Tool [7] and UNFPA Regional Survey templates used in reproductive health program monitoring in EECA countries [8,9]. The survey included a mix of structured, closed-ended questions covering the following parameters:

- Country/Territory;
- Introduction of HPV vaccination in the National Strategy;
- Introduction of HPV vaccination in the National Immunization Schedule;
- Year/month of introduction;
- Target age for females and males;
- Type of vaccine used;
- Number of doses;
- Coverage rates (latest dose, disaggregated by sex where available);
- Delivery methods (e.g., primary healthcare, schools, hybrid models).

The questionnaire underwent formal internal validation by UNFPA technical and regional experts to ensure clarity, content relevance, and usability across diverse country contexts. This process included expert review and iterative refinement based on feedback from initial pilot distributions in selected countries.

The final version was disseminated in November 2024 to national focal points from 17 countries and territories in the EECA region (for the purposes of this study, it refers to the group of 17 countries and territories included in the Regional Alliance framework).

Bosnia and Herzegovina were represented by three administrative entities (Federation of BiH, Republika Srpska, and Brčko District), resulting in 19 unique submissions.

Responses were coordinated through UNFPA Country Offices. Data represent policy-level summaries; specifically, the responses reflect aggregated national information provided by designated health ministry focal points, summarizing official program parameters, target populations, vaccine schedules, and reported coverage estimates. No raw or disaggregated data from individual vaccine recipients were collected as part of this study. However, the country data were self-reported by national stakeholders and were not independently verified; this is a limitation of the study.

Descriptive statistics were used to analyze responses. In addition, exploratory inferential analyses were conducted to assess associations between program characteristics and vaccination coverage. These included:

- One-way ANOVA and Kruskal-Wallis tests to compare mean female vaccination coverage across different vaccine types (Gardasil 4, Gardasil 9, and mixed use);
- Chi-square tests to examine associations between delivery model (e.g., Primary Health Care (PHC) vs. school-based) and high (>70%) versus low (<70%) coverage;
- Spearman correlation to explore potential relationships between policy maturity and coverage levels. In particular, we defined policy maturity as the number of years elapsed since a country initiated its HPV vaccination program, as of 2024. Based on this, countries were grouped into three categories: early adopters (programs launched before 2021), mid-phase adopters (2021–2023), and new adopters (2024 onward).

Vaccination coverage was defined as the percentage of the eligible target population that had received the full HPV vaccine dose regimen by the time of data collection [10].

Statistical analysis was performed using IBM SPSS Statistics for Windows, Version 29.0 (IBM Corp., Armonk, NY, USA). Assumptions of normality and homogeneity of variances were assessed. When ANOVA results were significant, Tukey’s post hoc test was performed, and effect sizes (partial η^2^) were reported.

A Chi-square test with a contingency table was used to evaluate the association between delivery platform (PHC, school-based, hybrid) and vaccination coverage categorized as high (≥70%) or low (<70%). Statistical significance was set at *p* < 0.05.

## 3. Results

All the countries and territories—comprising 17 countries/territories and the three administrative units of Bosnia and Herzegovina—submitted complete responses to the questionnaire, ensuring full regional representation in the analysis (Table 1 and Table 2).

As of November 2024, six countries had launched their national HPV programs before 2021 (Armenia, Georgia, Moldova, North Macedonia, Turkmenistan, Uzbekistan), while six others (Albania, Bosnia and Herzegovina, Kazakhstan, Kosovo (UNSCR 1244), Kirghizstan and Serbia) initiated programs after the formation of the Alliance. Vaccination coverage among girls ranged from under 5% in some Bosnian entities to nearly universal coverage in Uzbekistan (99.3%) and Turkmenistan (98.5%).

Most programs adhered to WHO recommendations targeting girls aged 9–14, while others—like Armenia and Georgia—extended eligibility up to 45 years for girls and 26 or 45 years for boys, reflecting catch-up vaccination strategies. Male inclusion was observed in nine countries.

In terms of vaccine use, Gardasil 4 remained the most widely used across the region, although Gardasil 9 adoption has accelerated, particularly among newer programs. Delivery platforms also varied: 16 of the 19 implementations used primary healthcare as the primary channel, with several integrating school-based platforms to enhance reach.

### 3.1. Coverage Rates and Gaps

Among countries with active programs, coverage varies widely. Albania reports the highest recent uptake with 75.5% of 13-year-old girls receiving at least one dose. North Macedonia (58.8%) and Kirgizstan (63%) also show significant advancements in vaccination coverage. Armenia’s broader program, despite a long-standing start, achieves only 33.7% coverage as of 2024 projections. Bosnia and Herzegovina show early stage uptake, ranging from 0.2% to 4.2% among girls and up to 2.3% among boys in RS.

**Figure 1:**
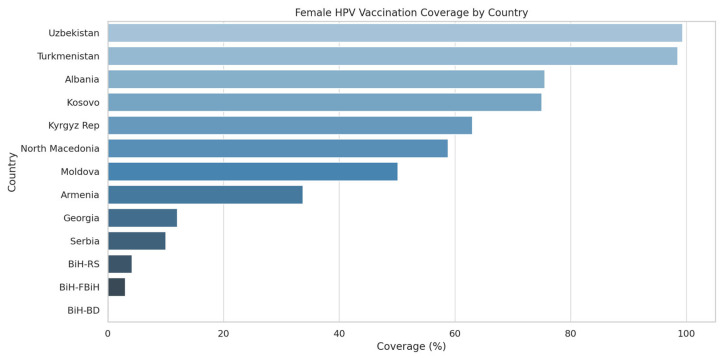
Female HPV Vaccination Coverage per Country.

Several countries—Turkmenistan and Uzbekistan—are regional leaders, having reached over 90% full vaccination coverage among girls by age 15, thus meeting the WHO’s 2030 target on this pillar years ahead of schedule (Figure 1).

**Figure 2:**
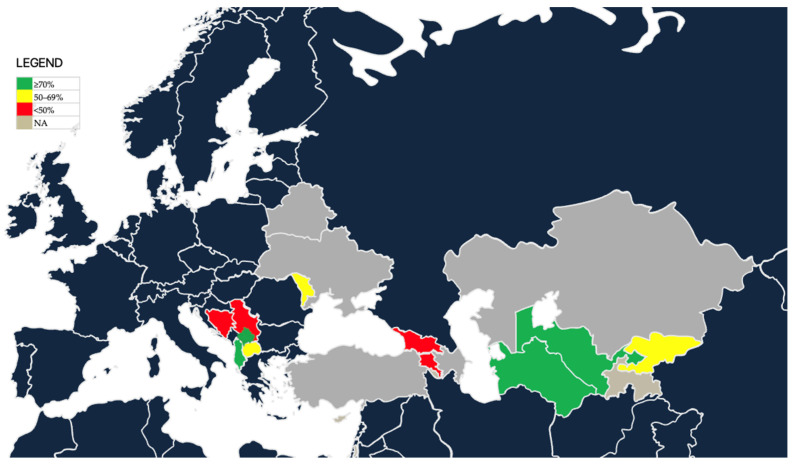
Regional distribution of HPV vaccination female coverage in EECA countries (as of November 2024).

A user-oriented geographic representation of national estimates of female HPV vaccination coverage is presented (Figure 2). Countries are categorized as high coverage (≥70%, green), moderate coverage (50–69%, yellow), low coverage (<50%, red), or no programme/no available data (grey).

### 3.2. Target Populations and Gender Inclusion

**Figure 3:**
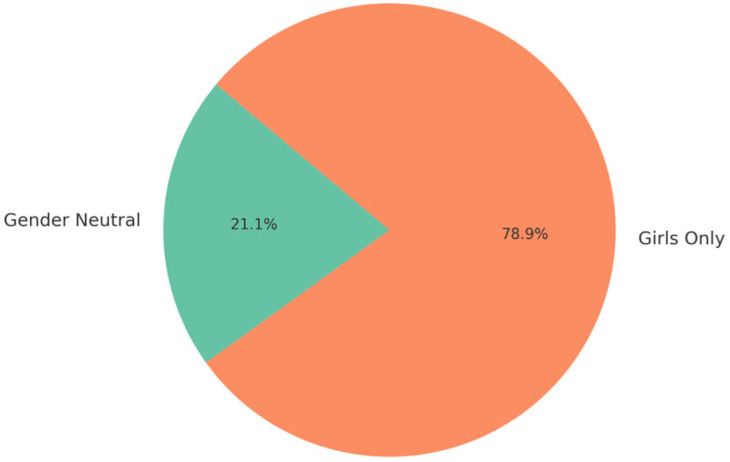
Gender inclusion.

Country data reveals significant heterogeneity in the definition of target populations. Albania, FBiH, and BD have a primary focus on 13- to 14-year-old girls, aligning with WHO recommendations, providing additional expanded options. Georgia (13–45 years) and Armenia have the wider age window (14–45) for girls, reflecting attempts to implement catch-up vaccination in adults. Several countries/territories like Moldova and Serbia include both girls and boys of birth cohort 9–14, showing a gender-inclusive implementation (Figure 3).

### 3.3. Vaccine Type and Schedule

**Figure 4:**
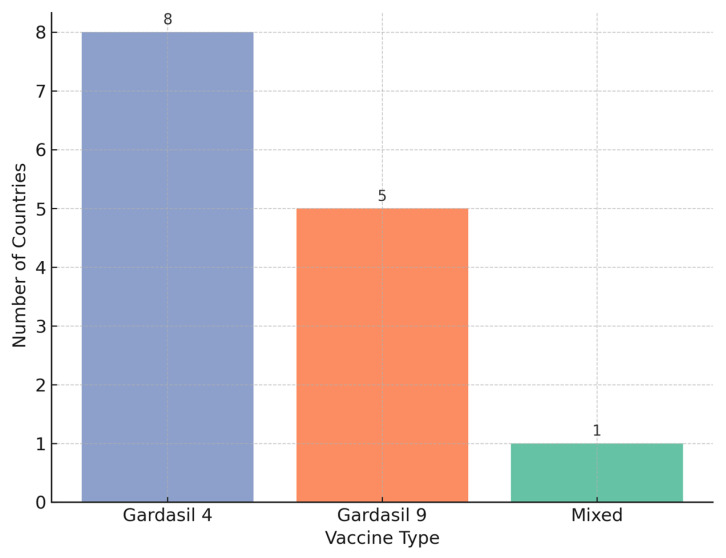
HPV Vaccine Types Used in National Programs.

The choice of vaccine also reflects a regional trend (Figure 3). Earlier adopters like Armenia and Albania initially used Gardasil 4, while more recent introductions such as those in Bosnia and Herzegovina have opted for Gardasil 9, in line with WHO’s 2022 updated guidance favoring broader coverage against high-risk HPV types. The two-dose schedule (0/6 months) is standard across all programs, enhancing feasibility and compliance, with Albania and Kosovo (UNSCR 1244) being the only countries so far reporting a single-dose initiation. A visual summary of the regional vaccination coverage per vaccine type is provided in Figure 4.

### 3.4. Delivery Platforms

**Figure 5:**
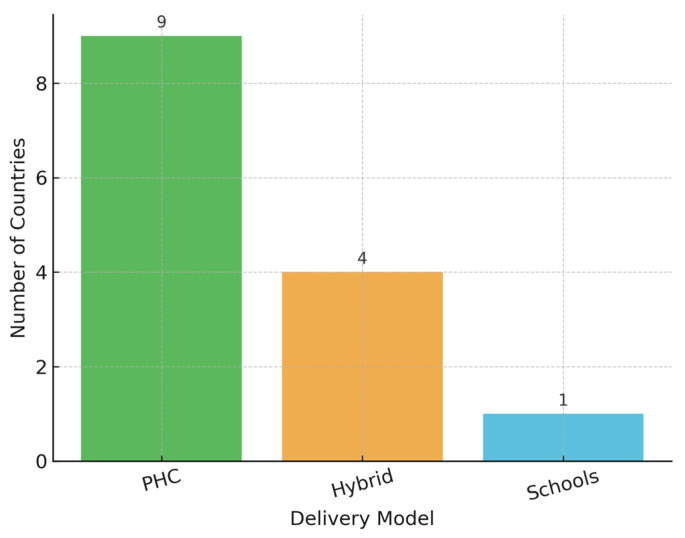
Delivery platforms.

Most countries rely on primary healthcare (PHC) systems for vaccine delivery (Figure 5). Belarus and Turkmenistan combine school-based and PHC platforms, a hybrid approach known to improve access among adolescents. In Azerbaijan, where no national public program exists, the vaccine is only available through private-sector channels, limiting equity and scale.

To further assess potential drivers of vaccine coverage variation, inferential statistical analyses were conducted. One-way ANOVA indicated significant differences in mean female vaccination coverage by vaccine type (F(2, 11) = 8.59, *p* = 0.0067), supported by a non-parametric Kruskal-Wallis test (H = 7.48, *p* = 0.0238).

Programs using Gardasil 4 reported significantly higher coverage rates compared to those using Gardasil 9 or a mixed schedule, suggesting that the choice of vaccine formulation may influence implementation effectiveness. However, while programs using Gardasil 4 reported significantly higher average coverage than those using Gardasil 9 or mixed regimens, this finding should be interpreted cautiously given the unequal group sizes and potential confounding factors such as program maturity and delivery infrastructure.

Additionally, a Chi-square test revealed a statistically significant association between delivery model and achievement of high vaccination coverage (χ^2^ = 8.83, *p* = 0.0121).

Countries employing school-based or hybrid delivery strategies more frequently reported coverage rates exceeding 70%. While this suggests a potential association between delivery platform design and coverage outcomes, the observational nature of the data precludes any inference of causality.

A Chi-square test of independence showed a statistically significant association between delivery model and achievement of high coverage (χ^2^(2) = 8.83, *p* = 0.0121). Countries using school-based or hybrid delivery strategies were more likely to report high coverage compared to PHC-only platforms. The contingency table results are presented in Table 3.

A Spearman correlation revealed a moderate positive association between policy maturity and female vaccination coverage (ρ = 0.52, *p* = 0.067). While the correlation did not reach conventional levels of statistical significance, the trend suggests that longer-established programs may achieve higher levels of coverage, likely due to greater institutional experience, community awareness, and infrastructure stability.

Overall, these results reinforce both the diversity and the progress of HPV vaccination efforts in the EECA region while underscoring the influence of vaccine type, delivery method, and policy maturity on national outcomes.

## 4. Discussion

i. Policy Impact

Country-level trends highlight how policy decisions and implementation timelines have influenced outcomes across the EECA region. Armenia, Georgia, Moldova, North Macedonia, Turkmenistan, and Uzbekistan launched HPV programs prior to 2021 and generally report moderate to high levels of coverage. Uzbekistan (99.3%) and Turkmenistan (98.5%) have already exceeded WHO’s 90% target, setting a benchmark for the region. North Macedonia (58.8%) and Kyrgyz Republic (63.0%) show steady progress following earlier implementation phases. Post-2021 adopters like Kazakhstan, Kosovo (UNSCR 1244), and Albania have made strong early gains, with Kosovo (UNSCR 1244) reporting 75% coverage in its initial year and Albania reaching 75.5% among 13-year-old girls. Ukraine has yet to implement a national program, while Belarus plans to initiate nationwide vaccination in September 2025. Tajikistan is also at the early phase of program launch. These country-specific experiences underscore the varying degrees of policy commitment, health system capacity, and regional coordination. A moderate positive correlation between policy maturity and vaccination coverage (ρ = 0.52, *p* = 0.067) further suggests that early, sustained policy engagement contributes to better outcomes over time.

The observed timing between the launch of national HPV vaccination programmes and the creation of the Regional Alliance 2021, during which several countries initiated or scaled up their strategy, suggests potential alignment of efforts; however, this should be interpreted as an exploratory observation.

The expansion of HPV vaccination programs across the region appears temporally aligned with the establishment of the Regional Alliance in 2021, during which several countries initiated or scaled up national efforts. While this temporal association may indicate a contributory role of the Alliance, definitive causal inference cannot be made in the absence of control groups or independent validation mechanisms. Nonetheless, the Alliance likely played a catalytic role by promoting cross-country technical dialogue and regional solidarity, which may have influenced policy momentum and harmonization [11,12,13,14].

ii. Equity

Significant inequities exist both between and within countries. While Uzbekistan and Turkmenistan have surpassed the 90% target, countries like the entities of Bosnia and Herzegovina—FBiH (3.0%), RS (4.2%), and BD (0.2%)—face major implementation hurdles. Armenia’s broader catch-up approach has yielded 33.7% coverage, whereas Georgia, despite earlier adoption, reports low uptake (12%). Moldova (50.1%) and Kyrgyz Republic (63.0%) represent mid-range performers, while Serbia remains below 10%. Albania has made rapid progress post-launch, while Ukraine and Belarus (pre-launch) continue to exhibit structural and policy gaps. These disparities reflect access limitations, delayed procurement, logistical fragmentation, and inconsistent political prioritization. Equity gaps also manifest within countries based on administrative fragmentation and uneven health service infrastructure [15]. Newer programs often lack the outreach mechanisms of more mature systems. Continued regional support is needed to ensure that lagging countries receive tailored assistance to close these gaps.

iii. Vaccine Access Models

Program success is strongly linked to delivery mechanisms. Countries employing hybrid school-PHC models report high or promising uptake, suggesting that reaching adolescents where they study improves coverage; however, this observation remains exploratory. Kyrgyz Republic and Moldova also leveraged PHC-based systems effectively. Kosovo (UNSCR 1244) adopted both school and PHC channels, achieving over 75% coverage in its first year. In contrast, Armenia and Georgia rely predominantly on PHC systems and show moderate-to-low results. These observations highlight the need for adaptable and multisectoral approaches tailored to national contexts, as delivery models and vaccine selection emerged as critical operational determinants of success. Indeed, countries using school-based or hybrid models were significantly more likely to achieve high vaccination coverage (χ^2^ = 8.83, *p* = 0.0121). Similarly, coverage varied by vaccine type, with Gardasil 4 associated with higher average uptake than Gardasil 9 or mixed-use schedules (ANOVA *p* = 0.0067). However, the observed association between higher coverage and the use of Gardasil 4 may reflect underlying programmatic factors—such as earlier adoption, stronger delivery systems, or national procurement patterns—rather than the vaccine formulation itself. Given the unequal distribution of vaccine types across countries, these results should be interpreted with caution. This is a limitation of the study.

iv. Gender Inclusion

The study confirms growing gender inclusivity in national HPV vaccination strategies. Nine countries and territories—Armenia, Georgia, Moldova, North Macedonia, Serbia, Kosovo (UNSCR 1244), BiH-RS, BiH-BD, and Turkmenistan—now include boys in their vaccination schedules, a reflection of increasing alignment with WHO’s gender-neutral guidance. Notably, Armenia offers vaccination for males up to age 45, while Georgia covers boys up to 26. Serbia and North Macedonia provide inclusive vaccination to boys aged 5–19 and 12–19, respectively. However, uptake among boys remains low and inconsistently reported in official statistics, particularly in newer or pilot-stage programs. Kosovo (UNSC 1244) and Turkmenistan appear to lead in early gender parity, while implementation remains pending or partial in other countries such as Albania and Kazakhstan. Expanding coverage among boys and ensuring reliable data collection by sex will be critical to achieving gender equity in immunization [16].

## 5. Limitations and Future Directions

This study has several limitations that warrant consideration. First, the analysis relies primarily on self-reported national data submitted by designated country focal points. These data were not externally validated and may be subject to recall bias, inconsistencies in data definitions, or overestimation of programme performance. To partially mitigate this, the authors cross-referenced country responses with secondary data sources, including WHO/UNICEF Joint Reporting Forms and UNFPA monitoring reports, where available; however, comparable independent data were not uniformly accessible across all EECA countries at the time of data collection (November 2024).

Second, the country-level sample size (N = 19) inherently limits statistical power and restricts generalisability. Exploratory inferential analyses (ANOVA and Chi-square) were introduced to offer preliminary insights; however, small group sizes (e.g., Gardasil 4 vs. Gardasil 9 adopters) and unequal distribution of delivery strategies should be considered when interpreting findings. No formal calculation of confidence intervals was possible within this design.

Third, by nature of its design, the study presents policy-level summaries, which may not fully capture intra-country or subnational variability in vaccine uptake.

In line with the descriptive nature of the study design and the data limitations, the observed associations are interpreted as exploratory correlations without inference of causality. The results are intended to inform hypothesis generation and identify areas for further empirical investigation rather than to establish definitive causal relationships.

Future studies combining longitudinal follow-up, systematic independent data verification, and subnational disaggregation would be critical to strengthen future evaluations. Notwithstanding these limitations, the study provides valuable comparative insights into HPV vaccination scale-up across the EECA region. The observed interplay between policy maturity, delivery platform choice, and equity outcomes highlights areas for technical assistance and regional cooperation to meet global cervical cancer elimination targets.

## 6. Conclusions

This study provides a regional overview of HPV vaccination implementation in Eastern Europe and Central Asia and highlights both achievements and ongoing challenges in progressing toward WHO Target 1. While several countries have achieved or are approaching 90% coverage, others remain at early rollout stages, facing structural, logistical, and policy-related barriers.

The findings underscore the importance of program design features, such as vaccine type and delivery model, in influencing outcomes [17]. School-based and hybrid delivery strategies, in particular, were associated with higher coverage. The role of policy maturity and sustained national investment also emerged as relevant, reinforcing the importance of long-term planning and institutional continuity.

While the Regional Alliance may have supported progress in several countries, its impact should be interpreted within a broader ecosystem of national and international efforts. Future initiatives should focus on building technical capacity, fostering cross-country learning, and ensuring equitable access to vaccination.

To accelerate progress and close coverage gaps, countries should consider the following priority actions:

- Prioritize school-based or hybrid delivery models where feasible;
- Expand vaccine eligibility to include boys and older adolescent catch-up cohorts;
- Ensure timely procurement and supply chain readiness;
- Strengthen national information systems for tracking and evaluation;
- Foster intersectoral collaboration across ministries and partners.

A shared regional commitment to these principles will be essential to achieving cervical cancer elimination as a public health problem by 2030.

## Figures and Tables

**Table 1:** HPV Vaccination in EECA Region (November 2024).

Country	Included in National Strategy	Included in Vaccination Calendar	Month/Year Started	Females (Target/Expanded Age Range)	Males (Target/Expanded Age Range)	Vaccine	Number of Doses (Schedule)	Coverage (Females-Last Dose)	Coverage (Males-Last Dose)	Delivery
Albania	Yes	Yes	Nov 2022	13 14–20	-	Gardasil 4	1	75.5% (Girls 13, 2023) ^1^	-	PHC
Armenia	Yes	Yes	Dec 2017	13 14–45	14 15–45	Gardasil 4	2 (0/6)	33.7% (Dec 2024) ^2^	-	PHC
Azerbaijan	No	-	N/A	-	-	-	-	-	-	Only private sector
Belarus	Yes	Yes ^3^	Sep 2025	11	NA	-	-	-	-	Hybrid
BiH-FBiH	Yes	Yes	Jan 2023	13–14	NA	Gardasil 9	2 (0/6)	3.0% (Oct 2024) ^4^	-	PHC
BiH-RS	Yes	Yes	Mar 2023	9–14	9–14	Gardasil 9	2 (0/6)	4.2% (Oct 2024) ^4^	2.3% (Oct 2024)^4^	PHC
BiH-BD	Yes	Yes	Sep 2024	9–14	9–14	Gardasil 9	2 (0/6)	0.2% (Oct 2024) ^4^		
Georgia	Yes	Yes	Sep 2019	10–12 13–45	10–12 13–26	Gardasil 4 (Gardasil 9 in 2024)	10–14: 2 (0/6) 15–45: 3 (0/2/6)	12% (Dec 2024) Girls aged 10–12	7% (Dec 2024) Boys aged 10–12	PHC
Kazakhstan ^5^	Yes	Yes	Sep 2024	11 12–13	-	Gardasil 4	2 (0/6)	-	-	Hybrid
Kosovo under UNSCR 1244	Yes	Yes	Oct 2023 (F)Feb 2025 (M)	12–13 ^6^	12–13	Gardasil 4	1	75% (2023) ^7^ 84% According to the latest country data	Q1 2025	Hybrid
Kyrgyz Rep	Yes	Yes	Nov 2022	11	-	Gardasil 4	2 (0/6)	63.0% (2023) ^8^	-	Schools
Moldova	Yes	Yes	Sep 2021	9–14	9–14	Gardasil 4	2 (0/6)	50.1% (2023) ^7^	-	PHC
North Macedonia	Yes	Yes	Oct 2009	12–19	12–19	Gardasil 9	12–14: 2 (0/6) 15–19: 3 (0/2/6)	58.8% (Jan-Jun 2024) 56.6% (2023) ^7^	Q2 2024	PHC
Serbia	Yes	Yes	Jun 2022	9–14 15–19	9–14 5–19	Gardasil 9	9–14: 2 (0/6) 15–19: 3 (0/2/6)	10.0% (Oct 2024) ^9^	3.0% (Oct 2024) ^10^	PHC
Tajikistan	Yes	Yes	Sep 2025	10	-	Gardasil 4	2 (0/6)	-	-	PHC
Türkiye	No	No ^10^	-	-	-	-	-	-	-	
Turkmenistan	Yes	Yes	Oct 2016	9	9	Gardasil 4	2 (0/6)	98.5% (2023) ^7^	98.5% (2023) **^1^**	Hybrid
Ukraine	Yes	No ^11^	-	-	-	-	-	-	-	
Uzbekistan	Yes	Yes	Oct 2019	9	NA	Gardasil 4	2 (0/6)	99.3% (11 months 2024, MoH)	-	Hybrid

^1^ Albanian Ministry of Health; ^2^ Armenian Ministry of Health; ^3^ The government of Belarus has included HPV vaccination for girls age 11 in the national vaccination calendar (MoH resolution #111 dated 1 July 2024); ^4^ Government of BiH, 1st dose; ^5^ Government of Kazakhstan (https://primeminister.kz/en/news/over-97-billion-tenge-allocated-by-government-for-human-papillomavirus-vaccine-procurement-28464 (accessed on 6 April 2025)); ^6^ To be expanded to females 12–21; ^7^ WHO data (https://immunizationdata.who.int/global/wiise-detail-page/human-papillomavirus-(hpv)-vaccination-coverage (accessed on 6 April 2025)); ^8^ Government of Kyrgyz Republic; ^9^ Estimated from the aggregated data available from the Institute of Public Health; ^10^ Three municipalities are piloting HPV vaccination and the Ministry of Health is undertaking a technical study for a national HPV vaccination program; ^11^ Ukraine is in the final stages of including HPV vaccination in the national vaccination calendar.

**Table 2:** HPV Vaccination Program Overview by Subregion and Detailed Vaccination Data.

Subregion	Country	Program Start Year	Vaccine Type	Delivery Model
Eastern Europe	Belarus	2025	NA	NA
Eastern Europe	Moldova	2021	Gardasil 4	PHC
Eastern Europe	Türkiye	NA	NA	NA
Eastern Europe	Ukraine	NA	NA	NA
South Caucasus	Armenia	2017	Gardasil 4	PHC
South Caucasus	Azerbaijan	NA	NA	NA
South Caucasus	Georgia	2019	Mixed	PHC
Western Balkans	Albania	2022	Gardasil 4	PHC
Western Balkans	Bosnia and Herzegovina (BD)	2024	Gardasil 9	PHC
Western Balkans	Bosnia and Herzegovina (FBiH)	2023	Gardasil 9	PHC
Western Balkans	Bosnia and Herzegovina (RS)	2023	Gardasil 9	PHC
Western Balkans	Kosovo (UNSCR 1244)	2023	Gardasil 4	Hybrid
Western Balkans	North Macedonia	2009	Gardasil 9	PHC
Western Balkans	Serbia	2022	Gardasil 9	PHC
Central Asia	Kazakhstan	2024	Gardasil 4	Hybrid
Central Asia	Kyrgyz Republic	2022	Gardasil 4	Schools
Central Asia	Tajikistan	NA	NA	NA
Central Asia	Turkmenistan	2016	Gardasil 4	Hybrid
Central Asia	Uzbekistan	2019	Gardasil 4	Hybrid
Country	Target Age (Girls)	Target Age (Boys)	Coverage Female (%)	Coverage Male (%)
Albania	13	None	75.5	None
Armenia	9–26	9–45	33.7	None
Azerbaijan	NA	NA	NA	NA
Belarus	NA	NA	NA	NA
Bosnia and Herzegovina (BD)	9–14	None	0.2	None
Bosnia and Herzegovina (FBiH)	9–14	None	3.0	None
Bosnia and Herzegovina (RS)	9–14	None	4.2	None
Georgia	9–45	9–26	12.0	None
Kazakhstan	9–14	None	None	None
Kosovo (UNSCR 1244)	9–14	None	75.0	None
Kyrgyz Republic	9–14	None	63.0	None
Moldova	9–14	None	50.1	None
North Macedonia	12–19	12–19	58.8	None
Serbia	5–19	5–19	10.0	None
Tajikistan	NA	NA	NA	NA
Turkmenistan	9–14	None	98.5	None
Türkiye	NA	NA	NA	NA
Ukraine	NA	NA	NA	NA
Uzbekistan	9–14	None	99.3	None

Notes: NA = Not available. PHC = Primary Healthcare. Mixed = use of both Gardasil 4 and Gardasil 9 within the national programme. Hybrid = delivery through both School-based programmes and PHC facilities.

**Table 3:** Contingency table of delivery model vs. achievement of high vaccination coverage.

Delivery Model	High Coverage (≥70%)	Low Coverage (<70%)	Total
School-based	5	2	7
PHC-only	1	5	6
Hybrid	4	2	6
Total	10	9	19

Note: PHC = Primary Healthcare Facilities. Data as of November 2024.

## Data Availability

Country level and WHO official data.
